# Supplementary material for: Causal Relationship between Aging and Anorexia Nervosa: A White-Matter-Microstructure-Mediated Mendelian Randomization Analysis
Source: Biomedicines. 2024 Aug 16;12(8):1874. doi: 10.3390/biomedicines12081874 (PMC11351342; doi:10.3390/biomedicines12081874)
Supplement: Supplementary file 1 [file biomedicines-12-01874-s001.zip › Supplementary Figure/Figure S9/Leaveoneout plot of MR analysis for ubm-b-1515 to AN.pdf]

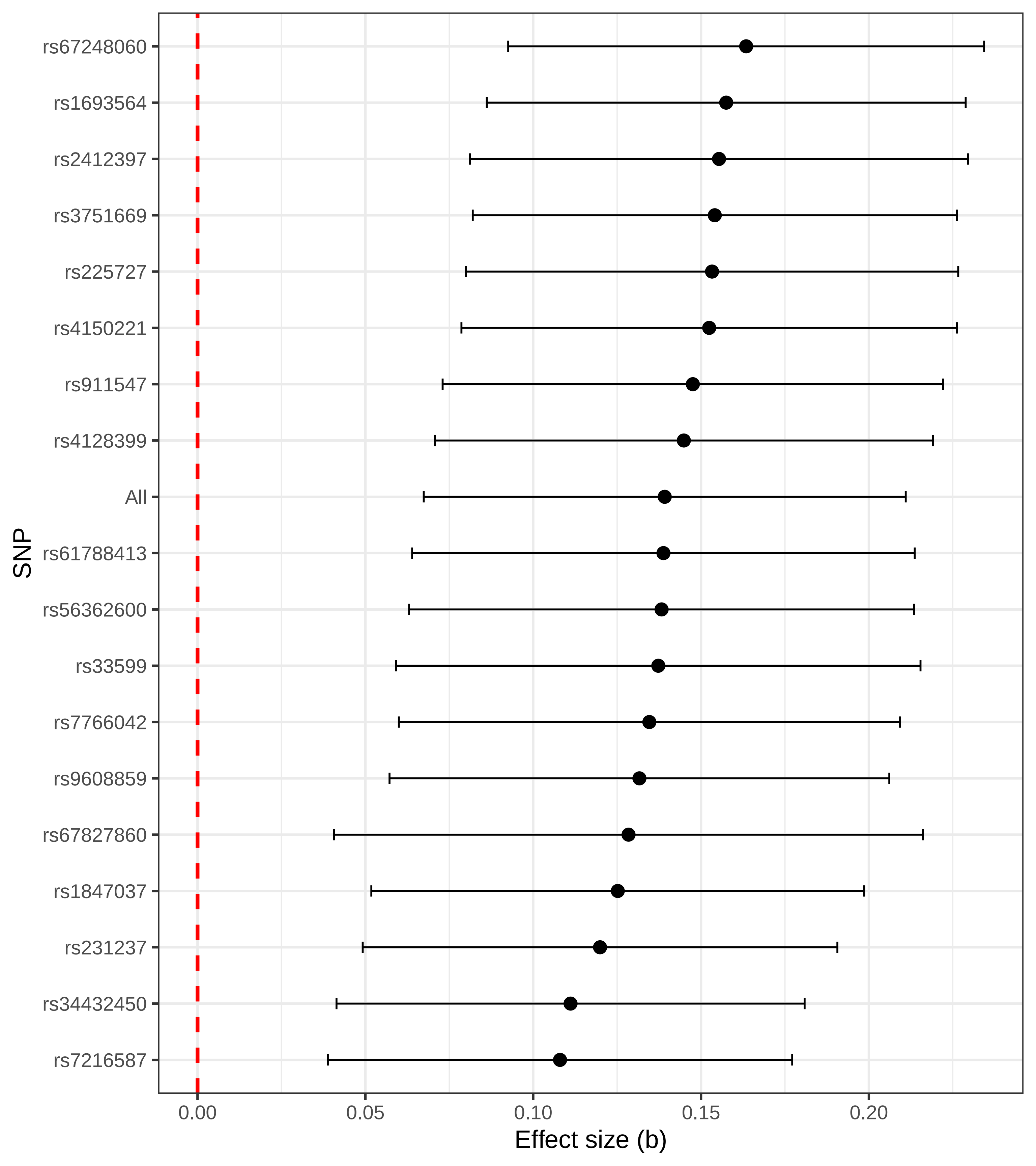
rs67248060
rs1693564
rs2412397
rs3751669
rs225727
rs4150221
rs911547
rs4128399
All
rs61788413
rs56362600
rs33599
rs7766042
rs9608859
rs67827860
rs1847037
rs231237
rs34432450
rs7216587
SNP
0.00
0.05
0.10
0.15
0.20
Effect size (b)
